# Dynamic Visual Acuity Results in Otolith Electrical Stimulation in Bilateral Vestibular Dysfunction

**DOI:** 10.3390/jcm11195706

**Published:** 2022-09-27

**Authors:** Isaura Rodríguez-Montesdeoca, Ángel Ramos de Miguel, Juan Carlos Falcón-González, Silvia Borkoski-Barreiro, Susana Benítez-Robaina, Gloria Guerra-Jimenez, Joana Pavone, Angel Ramos-Macías

**Affiliations:** 1 Department of Otolaryngology, and Head and Neck Surgery, Complejo Hospitalario Universitario Insular Materno Infantil de Gran Canaria, 35016 Las Palmas, Spain; 2 Hearing and Balance Laboratory, University of Las Palmas de Gran Canaria, 35001 Las Palmas, Spain; 3 Department of Otolaryngology, Faculty of Medicine, University of Las Palmas de Gran Canaria, 35001 Las Palmas, Spain

**Keywords:** bilateral vestibular disease (BVD), dynamic visual acuity (DVA), vestibulo-ocular reflex (VOR), oscillopsia severity questionnaire (OSQ), vestibular implant

## Abstract

(1) Background. Patients with bilateral vestibular disease (BVD) experience oscillopsia with a detriment to visual acuity (VA). This VA is driven mainly by the VOR that has two components: rotational and translational. VA can be tested by using dynamic visual acuity (DVA) on a treadmill because both systems are activated. The aim of this study is to compare VA before and after chronic electrical stimulation of the otolith organ. (2) Materials and Method. Five patients suffering from bilateral vestibular dysfunction (BVD), previously implanted with a new vestibular implant prototype, were included in this study with the aim to check VA with and without vestibular implant use (W and W/O) in static, 2 km/h and 4 km/h walking situations. DVAtreadmill was measured on a treadmill with a dynamic illegible E (DIE) test in static and dynamic conditions (while walking on the treadmill at 2 and 4 km/h). The DVA score was registered in a logarithm of the minimum angle of resolution (LogMAR) for each speed. In addition, every patient completed the oscillopsia severity questionnaire (OSQ) and video head impulse test (vHIT) before and after activation of the vestibular implant. (3) Results. The analysis shows a significant difference in OSQ scores and DVA with an improvement in dynamic conditions. Organized corrective saccades during the use of a vestibular implant with no changes in gain were also detected in the video head impulse tests (vHIT). (4) Conclusion. The vestibular implant with otolithic stimulation offers changes in the response of DVA, which makes this paper one of the first to address the possible restoration of it. It is not possible to rule out other contributing factors (presence of covert saccades, somatosensory system, …). More work seems necessary to understand the neurophysiological basis of these findings, but this implant is added as a therapeutic alternative for the improvement of oscillopsia.

## 1. Introduction

Patients with bilateral vestibular injuries experience an illusion of visual movement during head movements, known as oscillopsia, one of the main symptoms of the syndrome, which causes a reduction in their quality of life. Our movements in daily life have both translational and rotational components. In order to compensate for this situation, the central nervous system (CNS) keeps a stable image on the retina with compensatory movements, mainly driven by the vestibulo-ocular reflex (VOR) [1]. The retinal slippage, representing the degree of movement of the image through the retina, should not exceed 2°/s, to avoid VA loss [2]. According to the type of head movement, two types of VOR are distinguished: the angular VOR (aVOR), which responds to the angular acceleration detected by the semicircular canals (CSC), and the translational VOR (tVOR), related to the linear movement detected by the otoliths [3].

The aVOR generates compensatory eye movements for head rotation in both vertical and horizontal planes during slow movement and head accelerations up to 10 Hz or 4000°/sec^2^, but with the increase in the speed and frequency of head movements, the translational VOR (tVOR) is recruited to maintain gaze generating eye movements during head translation [4]. Although this tVOR has not received as much attention because the equipment to induce controlled and repeatable passive stimulation is expensive, we now know some aspects: at 2 Hz and at near distances, the gain of the compensatory tVOR is significantly increased [5] and there are decreases in the conjugate retinal image slip of the near target versus a distant background with the goal of minimizing the relative motion of one with respect to the other, and the binocular disparities during active or passive movements are minimized [6]. These eye movements complement and work synergistically with visuo-motor reflexes (ocular following reflex) [7].

Visual acuity can be tested by various functional vestibular tests such as the dynamic visual acuity (DVA). All DVA testing protocols evaluate horizontal and/or vertical semicircular canals in active or passive head movements. However, when walking, all vestibular sensors are stimulated indirectly, especially the vertical canals (head tilt movement) and otoliths (rebound—movement of the head up and down). The DVA test on a treadmill [8,9] involves head movements in the vertical plane at relatively low speeds (maximum peak 30°/s) and at a relatively low frequency (approximately 2 Hz). It has been established in previous studies that, at all walking speeds, it reaches frequency values of 2 Hz and this figure or higher is necessary to establish an exclusive gaze stabilization of the vestibular system without the influence of other oculomotor systems [3,10].

Although today there is no definitive solution for patients with bilateral vestibular dysfunction, some teams around the world have recently generated attempts to artificially restore the functionality of the vestibular system through implants that perform electrical stimulation of the vestibular afferent pathway, as reflected in previous articles.

Our research team, as reflected in previous articles, has developed a vestibular implant that performs direct stimulation on the otolithic organ, having proven benefits and promising results in various vestibular tests: Dizziness Handicap Inventory (DHI), Dynamic Gait Index (DGI), vestibular evoked myogenic potential (VEMPS), Subjective Visual Vertical (SVV), and posturography [11]. Based on these results, the present study aims to find out if there is an effect on oscillopsia after performing this stimulation. For that, oscillopsia was quantified objectively by dynamic visual acuity on a treadmill, approaching a physiological situation [8,11,12] and subjectively by the oscillopsia severity questionnaire (OSQ).

## 2. Patients and Methods

Five patients were included in this study with bilateral profound hearing loss and BVD according to Criteria Consensus of the Classification Committee of the Bárány Society and had received a new research vestibular implant (VI) [13]. The exclusion criteria for this study comprised: being unable to provide consent personally, not matching cochlear implantation criteria, ossification or other inner ear anomalies that prevent full insertion of electrodes, retrocochlear or central origins of hearing impairment, medical contraindications for surgery, chronic depression, dementia and cognitive disorders, cerebellar ataxias, downbeat nystagmus syndrome, peripheral neuropathies, Parkinson’s disease, atypical Parkinson’s syndromes, multiple system atrophies, central gait disorders due to normal pressure hydrocephalus, frontal gait disorders, lower-body Parkinson, subcortical vascular encephalopathy or multiple sclerosis.

**Figure 1:**
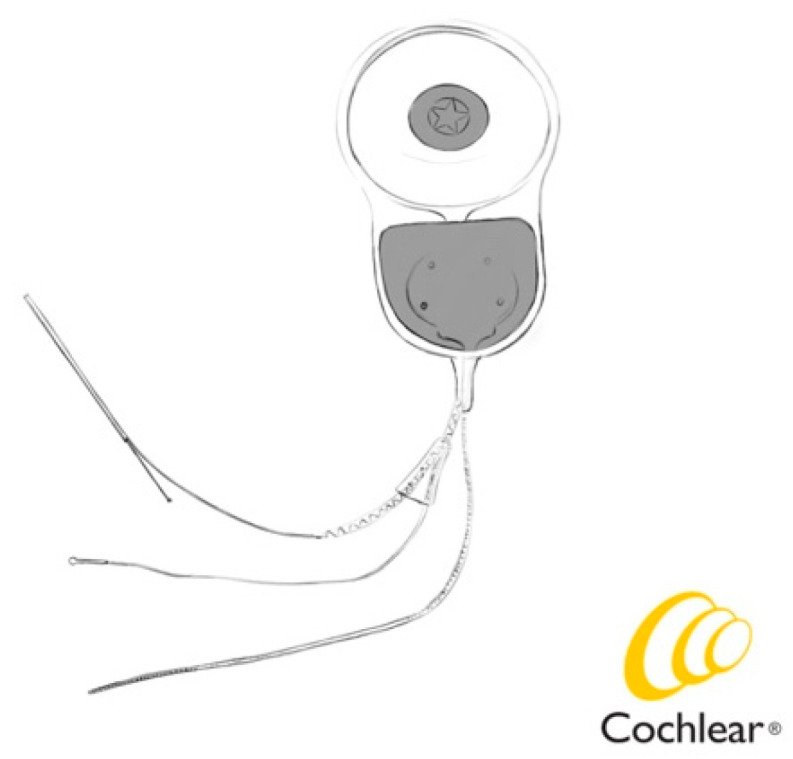
Cochleo-vestibular implant CI24VEST and external processor.

The vestibular implant is a custom-modified cochlear implant with two arrays: a full-band straight electrode, CI24RE(VEST), from Cochlear Ltd. (Cochlear Ltd., Lane Cove, NSW, Australia) with three active electrodes for VI stimulation (Figure 1). Full-band electrodes were selected to assure that the electrodes were facing the closest area of neural tissue related to the saccular area. For the cochlear stimulation, a perimodiolar array with 19 active electrodes (Cochlear Ltd., Sydney, NSW, Australia) was used in all of them.

All test were performed one month after activation. Patients used the implant every day, and except for sleeping hours patients used the implant between 8 and 16 h per day.

### 2.1. Surgery

**Figure 2:**
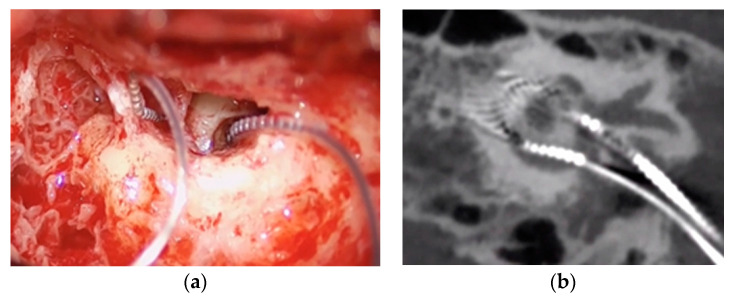
(**a**) Surgical image of electrodes’ position; (**b**) CT postoperative electrode position.

The same surgeon performed all procedures (A.R.M.). Enlarged retroauricular approach was performed. Then, after identifying the temporalis muscle, a flap was developed following the same principles as in standard CI surgery. As cochlear implantation was performed simultaneously, posterior tympanotomy was performed at this time with a clear exposure of the long process of incus, stapes and oval window. CI was inserted first. Once it was inserted and tested, VI was inserted. Opening of the vestibule was performed by performing a 0.5 mm stapedotomy medial and inferior to the anterior crura of the stapes in order to reach the closest area to inferior vestibular nerve afferent near the saccule macula, inserting the 3 first contacts of the vestibular component (Figure 2a).

During the surgical procedure, the facial nerve was monitored with the Nerve Integrity Monitor 2tm system (Medtronic, Minneapolis, MN, USA). Fixation of both electrodes was made independently. A vestibular response telemetry (VRT) was carried out intraoperatively (Python Software Foundation, version 2.4, Wilmington, DE, USA) in order to obtain electrically evoked action potentials (ECAPs) from the vestibular nerve [11].

During the postsurgical stay, CT scans and 3-D reconstruction were performed to check the placement and orientation of both electrodes (Figure 2b).

### 2.2. DVAtreadmill

DVA was assessed on a treadmill [14] (Domyos RUN 100), which includes a safety key to stop in case of a fall, with a screen placed at 2.8 m from the subject. The test was performed in front of a dynamic illegible E (DIE) test in order to repeat test without memorization [15]. The VA scale was adapted to the test subject distance, which was 2.8 m for all measurements. The chart was positioned at eye height. Patients were tested in static condition (while standing still) and in dynamic conditions (while walking on the treadmill at 2 and 4 km/h) with the vestibular implant turned on or off. Testing started with optotypes presented at a LogMAR of 1.0. When all optotypes were recognized correctly, the corresponding LogMAR was considered achieved and the last one obtained corresponded to the DVA score. A patient with a score of 1 or more line differences between static and dynamic acuity was defined as having an abnormal DVA. Every condition was tested once and a rest period of 1 min was inserted between each condition while the treadmill was stopped. If the patient was unable to keep a certain speed, the test was stopped and registered as “drop-out”. The speed of letters per row in each condition was calculated, as well as the decrease in LogMAR between static and dynamic conditions. DVAtreadmill was considered abnormal when a VA difference of more than 1 line was recorded at static, 2 and 4 km/h with and without vestibular implant.

When carrying out the test, it is essential to always use the same conditions, regarding the reading distance, brightness and ametropia correction. If the patient wears lenses (contact or glasses), the examination is performed with them, except in cases of progressive lenses where the test is performed without correction

### 2.3. Oscillopsia Severity Questionnaire (OSQ)

Every patient completed the oscillopsia severity questionnaire (OSQ) developed by the Division of Balance Disorders in Maastricht [8]. The OSQ includes nine questions about the patients’ experience of oscillopsia in daily life. Every question can be answered by one of the following five options: Always (=5), Often (=4), Sometimes (=3), Seldom (=2) or Never (=1). The outcome of every separate question was registered and the mean value for every patient was calculated. A mean value of three or more was considered as moderate to extreme oscillopsia severity.

### 2.4. VHIT

We also measured horizontal angular VOR gain and saccades by vestibular head impulse test (VHIT) (ICS Impulse type 1085 from GN Otometrics A/S, Taastrup, Denmark) with and without vestibular implant in the same visit to reduce the effect of learning over repeated trials. We also tested vertical canals, but we based our results only on horizontal ones because VOR gain of the verticals for the diagnosis of BVP has to be further evaluated [13].

### 2.5. Electrical Stimulation

The basic profile of electrical stimulation to obtain the vestibular response consists of an ACE (RE) coding strategy with MP1+MP2 stimulation, a maximum of 8, with a stimulus speed between 900 and 1200 Hz and a pulse width of 25 µs, depending on the patient’s response characteristics. Electrodes 1, 2, and 3 were used with the same C value and a dynamic range of 1, based on responses obtained intraoperatively.

In the patients, activation was achieved when reaching 80% of the intraoperative response value.

The programming of the cochlear part was carried out as established by the usual protocols 4 weeks after surgery, using 19 electrodes in this case. As a fitting method we use both electrodes’ arrays alternatively. After that, patients use both electrodes simultaneously for chronic electrical stimulation.

### 2.6. Ethical Considerations

This study was conducted in accordance with the guidelines contained in the Declaration of Helsinki on Ethical Principles for Medical Research Involving Human Subjects. This work was approved by the Ethical Committee of our hospital (Id: CEIM 2017/956, CEIM 2020-020-1-). The patients/participants provided their written informed consent to participate in this study.

Data were analyzed using IBM SPSS Statistics for Windows, Version 25.0. Armonk, NY: IBM Corp. Significance was set on *p* < 0.05. Scores of each item of the oscillopsia severity questionnaire were compared for patients with and without vestibular implant using Kruskal–Wallis test (IC 95% *p* < 0.05 for all comparisons). VA registered in logMAR and timings in each condition were also measured with a Student *t*-test (*p* < 0.05 for all comparisons).

## 3. Results

In the present study, five patients, all males with BV, were included. The mean age was 45 years (SD 9.41). The duration of illness varied between 5 and 20 years. Etiologies included Cogan syndrome, head trauma, bilateral Meniere disease, cholesteatoma and meningitis (Table 1).

In the present study, the subjects were used as their own controls so that the results with and without the implant were compared in the same visit to reduce a learning effect, and thus analyze if immediate changes were produced.

Four patients were able to complete the procedure at their own maximum safe walking velocity (2–4 km/h), and only one (Patient 1) was not able to complete 4 km/h with and without the vestibular implant. Absolute VA values obtained in each condition are presented in Table 1. VA differences between static, 2 and 4 km/h conditions were only normal in two patients (Patients 4 and 5) who had a normal VA difference with the vestibular implant at all three velocities (no more than one line of differences between the static and dynamic conditions). Most of the subjects were tested at 6 km/h as well, but we decided to reduce the examinations to just 4 km/h in order to prevent the falling risk for some of them.

**Figure 3:**
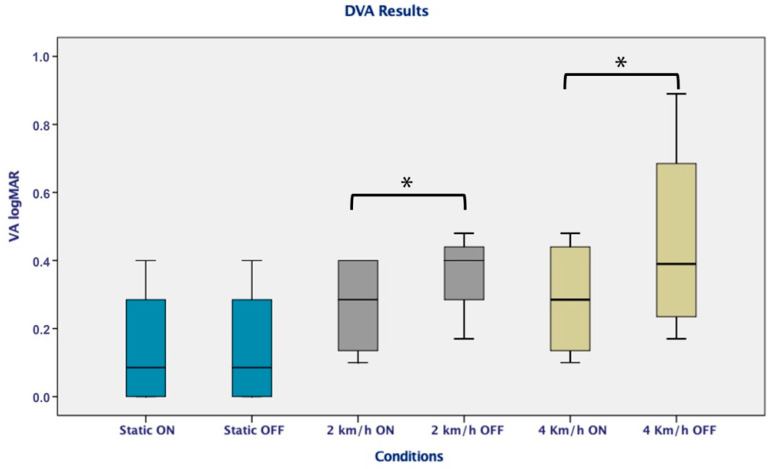
DVA on a treadmill with the vestibular implant on and off reflecting a significant difference in logMAR on 2 km/h and 4 km/h condition (*p* < 0.05). * Indicates significant differences between conditions.

**Figure 4:**
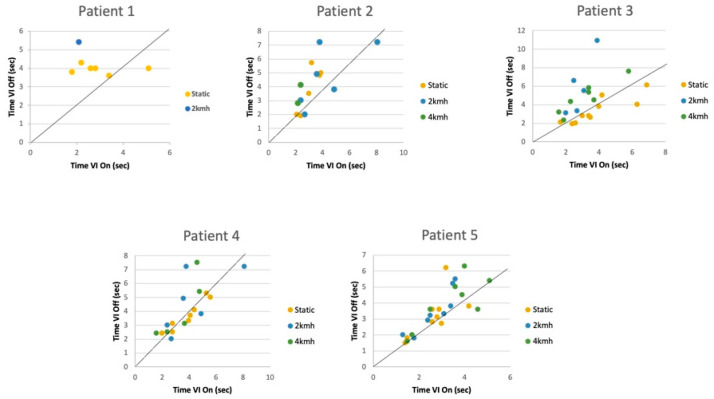
Timings per row for static and 4 km/h conditions in DVA on a treadmill with vestibular implant on and off in each patient. At 2 and 4 km/h, significant differences were acquired (*p* < 0.05).

Moreover, changes were evident during the DVA on the treadmill with the use of the implant, reflecting significant differences in logMAR with the vestibular implant on and off at 2 (*p* = 0.048; *p* = 0.043) and 4 km/h (*p* = 0.014; *p* = 0.033) (*p* < 0.05) (Figure 3). An analysis of the time per row to answer each line was also carried out, and a significant improvement (*p* < 0.05) resulted from the vestibular implant being on in the dynamic conditions, especially for the most difficult situation (4 km/h) (Figure 4)

The comparative study of OSQ results shows that the mean in most of the items is greater than or equal to 3 (8/9) and the result is reduced in the condition with the vestibular implant on. Significant differences were found using the patients themselves as controls (*p* < 0.05) (Table 2).

**Figure 5:**
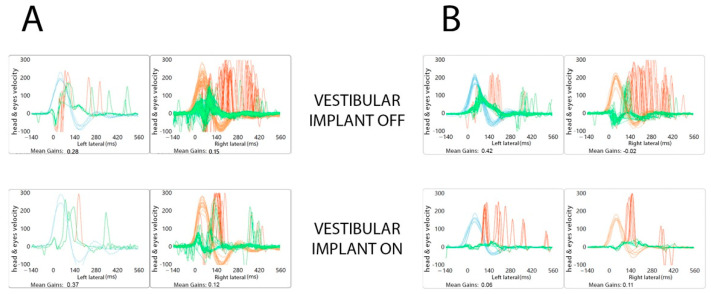
VHIT with an immediate saccadic reorganization process obtained in two of the patients (**A**,**B**).

Regarding the VHIT study of the five patients, a gain restoration was not achieved, although an immediate saccadic reorganization process was obtained in two of the patients (Patients 1 and 2) that suggests an absence in the stimulation in the six semicircular canals, as expected, but changes in VOR were reflected in VHIT with a substantial improvement in the DVA (Figure 5).

## 4. Discussion

In this research we wanted to quantify oscillopsia in BVD patients who use a new vestibular research implant CI24RE(VEST) and to compare the variations in VA with the device. We wanted to provide information on the otolithic involvement in the VOR since the role of the otoliths in humans (utricle and saccule) in the global vestibular function has not been widely studied and it is difficult to determine.

It is important to take into account that the subjects included in this study presented reduced vestibular activity before implantation, both at the otolithic organs and in the semicircular canals (tested by absence in VEMPS response, gain on VHIT < 0.4 and areflexia in caloric test), which ruled out the possibility of assuming outcomes in this study based on residual effects. In addition, no changes in VHIT gains were observed with the device, so it presumably indicates that the VA effect could be attributed to translational VOR.

One possible way to analyze reliable and sensitive VA in BVD patients is the evaluation of walking on a treadmill at controlled velocities because with frequency ranges around 2 Hz the VOR contribution in gaze stabilization is predominant [3,10]. Guinand et al. demonstrated a rise in test sensitivity for BVD by up to 97% by combining the three speeds of 2, 4 and 6 km/h (8). However, it should be taken into account that in patients with BVD, it has been described that up to 22% were not able to complete the DVA test on a treadmill (drop-out) [9], which coincides with our results since in one of the patients it was not possible to reach a speed of 4 km/h. The results that have been achieved in this study are promising; however, visual acuity that is considered normal has not been achieved at the moment, which can be justified by multiple factors, although one of them could be the lack of restoration of a VOR.

However, we must bear in mind that the DVA is a functional outcome of the multisensory system. A central processing of visual, vestibular and oculomotor inputs exists, and patients can use adaptation and compensation mechanisms to improve gait or gaze [16]: saccades, the vestibulo-collic reflex, automatic spinal locomotor programs [17], compensatory walking strategies with a reduction in walking speed or stride length [18], and that somatosensory system [7].

Although the aim of our implant was not the analysis of semicircular canals, interesting findings were obtained during the analysis of vHIT. In some of the patients, a reorganization of the saccades was observed on both sides when using the device, a finding that did not present when we repeated the test with the vestibular implant off. Recovery saccades with shorter latency would have resulted in the target landing on or near the fovea before it was extinguished, and this may have allowed for better DVA performance. [1,19,20]. This suggests that the reorganization of the refixation saccades influences the amount of vestibular disability [14]. The lack of improvement in VHIT gain and these saccades can represent the activation of a central compensating mechanism. The studies established so far have allowed theorizing that the constant electrical stimulation generates a barrage of action potentials in the saccular nerve, which replaces the reduced or absence of saccular afferent action potentials in these patients with BVD [21]. Theses action potentials are continuously arriving at the vestibular nuclei and the midline of the cerebellum and other structures, but we still do not know completely where saccular afferents are projected. These new pieces of information obtained in some of our patients, allow us to speculate that these central projections are not limited exclusively to motor control systems but are also associated with corrective saccades.

On the other hand, the OSQ of our patients shows similar results to those of Guinand 2012 [8]. Before using the implant, in static conditions, the subjects presented oscillopsia, even in the patient with the longest evolution of the disease (20 years) for whom it was supposed that he would have established central adaptation mechanisms for the tolerance of the retinal slip. However, this situation improved from extreme to moderate or soft oscillopsia severity with the use of the implant in many of the items. We consider this sensation of a certain degree of oscillopsia to be the result of an absence of a VOR restoration.

These findings in the DVA on a treadmill, coupled with the OSQ and the appearance of corrective saccades in the VHIT, make us assume that the inputs generated by the vestibular implant through the afferent pathways act on the central nervous system favoring a response that improves the adaptation and compensation mechanisms, and ultimately improving the patient’s visual acuity and thus their quality of life.

Despite of the lack of DVA test specificity, it is one of the few tests that evaluates the function of the otolithic organs close to reality and its widespread use allows us to perform comparatives with other research groups. We therefore hope with this research to provide information on vestibular stimulation, and the knowledge will be the start of continued research in the field with many unknowns still to be resolved.

## 5. Conclusions

The vestibular implant with otolithic stimulation offers changes in the response of the DVA, which makes this research one of the first to address the possible restoration of the tDVA, although it is not possible to rule out other contributing factors (presence of covert saccades, somatosensory system, …). More work seems necessary to understand the pathophysiology of these findings, but this implant is added as a therapeutic alternative for the improvement of oscillopsia.

## Figures and Tables

**Table 1:** We describe in this table demographic characteristics of patients and the DVA on a treadmill result in LogMAR with vestibular implant on and off in each condition.

Patient	Etiology	Sex	Age (Years)	Evolution (Years)	Years of Implantation	VA STATIC	VA 2 km/h	VA 4 km/h
P1 ON	Meningitis	Male	49	5	2018	0.40	1.00	
P1 OFF						0.40	1.00	
P2 ON	Cogan Syndrome	Male	47	6	2020	0.40	0.40	0.40
P2 OFF						0.40	0.40	0.89
P3 ON	Trauma	Male	48	20	2021	0.17	0.40	0.40
P3 OFF						0.17	0.40	0.48
P4 ON	Meniere Syndrome	Male	36	15	2021	0.00	0.10	0.10
P4 OFF						0.00	0.48	0.30
P5 ON	Cholesteatoma	Male	64	8	2022	0.0	0.10	0.10
P5 OFF						0.0	0.17	0.17

**Table 2:** Oscillopsia severity questionnaire with vestibular implant on and off with significant differences *p* < 0.05 in almost all the items (6/9). * Indicates significant differences.

Oscillopsia Severity Questionaire					
	Vestibular Implant Condition	N	Media	SD	SIG
OSQ ITEM 1	VI OFF	5	4.60	0.894	0.09
	VI ON	5	3.20	1.483	
OSQ ITEM 2	VI OFF	5	5.00	0.000	0.1
	VI ON	5	4.40	0.894	
OSQ ITEM 3	VI OFF	5	4.80	0.447	0.04 *
	VI ON	5	3.00	1.581	
OSQ ITEM 4	VI OFF	5	5.00	0.000	0.005 *
	VI ON	5	2.00	1.000	
OSQ ITEM 5	VI OFF	5	4,20	1.789	0.04 *
	VI ON	5	3.00	1.581	
OSQ ITEM 6	VI OFF	4	5.00	0.000	0.03 *
	VI ON	4	4.00	1.414	
OSQ ITEM 7	VI OFF	5	5.00	0.000	0.05 *
	VI ON	5	3.60	1.673	
OSQ ITEM 8	VI OFF	3	5.00	0.000	0.2
	VI ON	2	4.00	1.414	
OSQ ITEM 9	VI OFF	5	5.00	0.000	0.04 *
	VI ON	5	3.40	1.673	

## Data Availability

Data are available upon request to the corresponding author.
